# Expanding the phenotypic spectrum of familial digital arthropathy-brachydactyly due to a novel monoallelic variant in *TRPV4*: A report of the fourth family

**DOI:** 10.1016/j.gendis.2026.102158

**Published:** 2026-03-21

**Authors:** Xingzhu Geng, Yunyun Zhang, Chunhai Gao, Kai Liu, Yuda Wei, Xiaxia Liu, Hongyan Xu, Xiangyu Zhao

**Affiliations:** a Department of Laboratory Medicine, Key Laboratory for Laboratory Medicine of Linyi City, Shandong Provincial Medicine and Health Key Laboratory for Precise Diagnosis of Hereditary Rare Diseases, Linyi People’s Hospital, Shandong Second Medical University, Linyi, Shandong 276003, China; b Department of Medical Genetics, Linyi People’s Hospital, Shandong Second Medical University, Linyi, Shandong 276003, China

Familial digital arthropathy-brachydactyly (FDAB) is a rare autosomal dominant skeletal disorder caused by mutations in the *TRPV4* gene, characterized by shortened digits and progressive arthropathy. To date, only three *TRPV4*-related FDAB families have been reported worldwide. This study describes a new Chinese family with two affected individuals and provides a systematic analysis of their clinical presentation, radiological features, and genetic background. Both patients exhibited typical FDAB phenotypes consistent with previous reports, with the notable distinction that the proband presented with congenital brachydactyly in both hands. Whole-exome sequencing identified a novel heterozygous *TRPV4* variant, c.809G>C (p.Gly270Ala), which co-segregated with the disease phenotype. This study provides further evidence linking *TRPV4* mutations to the FDAB phenotype and expands the genotypic and phenotypic spectrum of *TRPV4*-related skeletal dysplasias (see Table 1).

**Table 1:** Mutations and clinical phenotypes of FDAB cases.

	Family 1	Family 2	Family 3	Family 4
Affected cases	16	5	1	2
Mutation (cDNA/protein)	c.819C>G (p.Phe273Leu)	c.812G>C (p.Arg271Pro)	c.809G>T (p.Gly270Val)	c.809G>C (p.Gly270Ala)
Location (domain)	ARD	ARD	ARD	ARD
Presentation at birth	Normal	Normal	Consistent with the FDAB as described	Both hands had brachydactyly, and the fourth toe of the right foot was short
Age of onset/site	In the first decade, hand changes	First decade, hand changes	Consistent with the FDAB as described	At birth, the hand changes
Progressive arthropathy in hands/feet	Present	Inflammation in the interphalangeal joints was apparent	Significant hand arthritis	Present
Body proportion	Normal	Proportional and similar to unaffected	Proportional relative to long bones	Normal
Peripheral neuropathy	Absent	Absent	Absent	Absent
Other phenotypes	Not explicitly mentioned	Not explicitly mentioned	Mild osteoarthritis of L3-L4-L5; mild bilateral medial and lateral compartment osteoarthritis of both knees	Absent
References	Lamande et al, *Nat Genet*, 2011; Amor et al, *Am J Med Genet*, 2002	Lamande et al, *Nat Genet*, 2011	Lamande et al, *Nat Genet*, 2011	This study

The clinical characteristics of FDAB were first systematically described and named by Amor et al in 2002,^1^ and were subsequently linked to mutations in the *TRPV4* gene (OMIM: 605427) in 2011.^2^ The most striking clinical hallmark of FDAB is the strict localization of pathological changes to the hands and feet, with no apparent impact on overall skeletal development or stature. The disease typically manifests in early childhood, with initial radiological findings often showing irregularity of the proximal articular surfaces of the distal interphalangeal joints, accompanied clinically by joint swelling, reduced range of motion, and relative shortening of the middle and distal phalanges. As the condition progresses, other interphalangeal, metacarpophalangeal, and metatarsophalangeal joints may become progressively involved, eventually leading to severe, painful, and deforming arthropathy.

This study involved four individuals across two generations, including two affected members (Fig. 1A). The proband is a 27-year-old female who presented with brachydactyly of both hands at birth, as well as shortening of the right fourth toe. Physical examination revealed shortening of the distal digits in both hands and feet, with the right fourth toe being particularly short. X-ray imaging showed shortened distal phalanges and narrowed joint spaces. The proband’s father also exhibited congenital brachydactyly in both hands and feet (Fig. 1B). Neither the proband nor her father showed spinal deformities or short stature.

**Figure 1:**
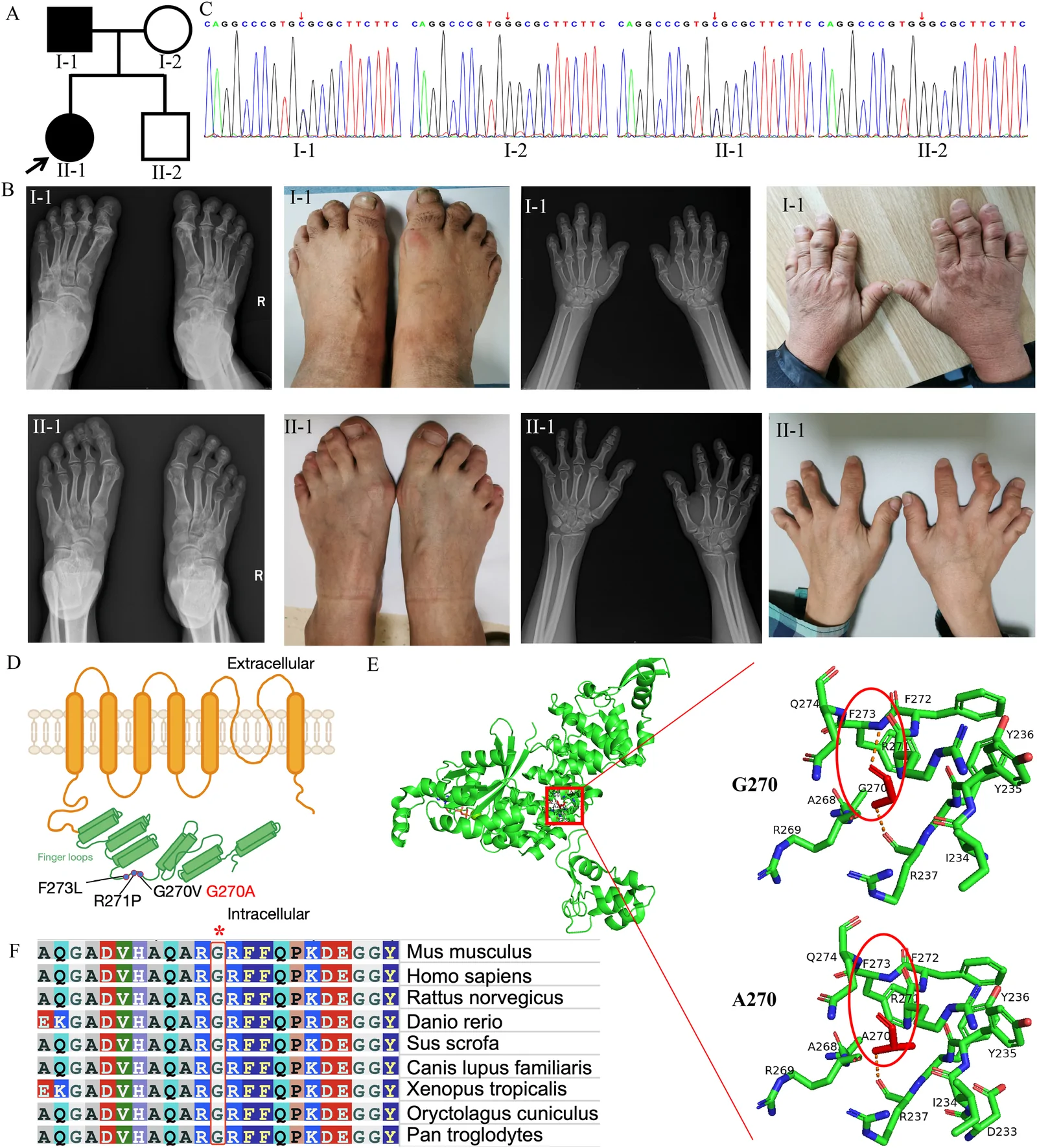
Clinical findings and genetic analysis. **(A)** Pedigree of the family with TRPV4 variants. **(B)** Photographs and radiographs of the hands and feet in the patients with the TRPV4 mutation. Both affected individuals showed distal brachydactyly in the hands and feet, accompanied by osteoarthropathy of the interphalangeal joints. The proband exhibited marked deformity of the fingers in the right hand and significant shortening of the fourth toe on the right foot. **(C)** Sanger sequencing confirmed that the proband (II-1) and her father (I-1) carried the mutation, while the other unaffected family members (I-2, II-2) did not carry this variant. **(D)** The location and schematic diagram of the mutation site within the predicted structure of the TRPV4 protein. **(E)** Structural modeling of wild-type and mutant TRPV4. Left: overall conformation of TRPV4 protein. Right: close-up view of residues within a 5 Å radius of p.Gly270 (upper) and p.Gly270Ala (lower). The central residues highlighted in red represent p.Gly270 and p.Ala270, respectively. The orange dotted lines represent hydrogen bonds between amino acids. The mutation of glycine to alanine introduces a side chain, which increases steric hindrance. In the wild-type structure, residue R270 forms two stable hydrogen bonds with F273 and R237, whereas in the G270A mutant, A270 retains only one hydrogen bond with R237, and the bond with F273 is completely lost. **(F)** Multiple-sequence alignment reveals that the amino acid at position 270 is highly conserved across multiple species.

Whole-exome sequencing analysis identified a novel heterozygous TRPV4 mutation (c.809G>C, p.G270A, NM_021625). Sanger sequencing confirmed its presence in the affected father and absence in unaffected family members (Fig. 1C), demonstrating genotype–phenotype co-segregation. This variant is located in the ankyrin repeat domain of the encoded protein. It occurs at the same position as the previously reported pathogenic mutation p.G270V but involves a different amino acid substitution (Fig. 1D). According to the American College of Medical Genetics and Genomics/Association for Molecular Pathology (ACMG/AMP) criteria, this variant is classified as pathogenic.

Through three-dimensional protein modeling, we found that the G270A mutation disrupts the local hydrogen-bonding network, particularly affecting its interaction with residue F273 (Fig. 1E). The collapse of this network suggests that the mutation severely compromises the structural integrity necessary to maintain the functional conformation of the “finger” loop. Notably, mutations at the key residue F273 have previously been reported to cause FDAB, further underscoring the functional importance of this region. Conservation analysis revealed that amino acids in this region were highly conserved across multiple species (Fig. 1F).

The *TRPV4* gene encodes a calcium-permeable cation channel, and its mutations can lead to a spectrum of skeletal and neurological channelopathies with diverse clinical manifestations, reflecting complex genotype–phenotype correlations and significant differences in the underlying pathogenic mechanisms.^3^ FDAB differs from other *TRPV4*-related skeletal dysplasias in that patients typically do not present with short stature, spinal deformities, or other systemic osteoarticular abnormalities. As with previously reported cases, the patients in this family exhibited aggressive arthropathy in the hands and feet, accompanied by shortening of the middle and distal phalanges. However, unlike in previous cases, where the phenotype often manifested gradually after birth, the proband in this study exhibited obvious brachydactyly in both hands and a shortened fourth toe on the right foot at birth, followed by progressively worsening arthropathy. This finding suggests that the clinical spectrum of TRPV4-related FDAB may be broader than currently recognized.

The TRPV4 protein is a membrane protein featuring six transmembrane domains, with an intracellular region composed of six ankyrin repeat sequences. Each repeat consists of two antiparallel alpha helices and a characteristic “finger” loop. To date, hundreds of pathogenic TRPV4 mutations have been reported. Of these, those associated with FDAB (such as F273L, R271P, and G270V) are located within the third finger loop, although this region also harbors mutations that cause other skeletal dysplasias or neuromuscular disorders. Damseh et al reported two patients from the same family carrying the G270V mutation who were diagnosed with Thiemann disease.^4^ Both FDAB and Thiemann disease are non-inflammatory digital arthropathies with overlapping phenotypes, affecting only the joints of the hands and feet; however, FDAB typically has an earlier age of onset. Thiemann disease mainly involves the proximal interphalangeal joints, with occasional toe joint involvement, and is characterized by avascular necrosis of the phalangeal epiphyses, as well as broad and short phalangeal metaphyses and epiphyses. In contrast, FDAB initially affects the distal interphalangeal joints. Notably, in their comparison of FDAB and Thiemann disease, Damseh et al reported that approximately 25% (5/20) of Thiemann cases exhibited clinical features consistent with FDAB. They proposed that the two conditions might represent different manifestations within the same disease, though further cases and genetic diagnostic evidence are still needed to confirm this hypothesis.

*TRPV4*-related disorders have traditionally been attributed to gain-of-function mutations, particularly those associated with severe skeletal dysplasias, such as certain forms of spondylometaphyseal dysplasia, or progressive peripheral neuropathies (CMT2C and SPSMA).^5^ Gain-of-function mutations typically induce sustained Ca^2+^ influx, leading to cytotoxicity and, consequently, severe systemic phenotypes. However, previous studies have shown that FDAB-associated mutations do not enhance channel activity. Rather, they disrupt the membrane trafficking of the protein, reducing TRPV4 surface expression and attenuating the response to activating stimuli.^2^ This partial loss-of-function may interfere, in a manner that is not yet fully understood, with the finely regulated calcium signaling that is essential for skeletal development and articular cartilage homeostasis. This ultimately results in the selective impairment of the distal phalanges and joints, without causing severe systemic defects.

A hallmark of FDAB is the restriction of pathological changes solely to the fingers and toes. Although it phenotypically shares common features with brachydactyly caused by mutations in classical brachydactyly genes like *IHH* and *GDF5*, the genetic backgrounds and molecular mechanisms involved are highly heterogeneous. Mutations in classical brachydactyly genes typically cause errors in developmental “blueprinting”, leading to pure and isolated skeletal shortening. In contrast, loss-of-function mutations in *TRPV4* are more akin to disrupting a “maintenance system” responsible for long-term joint integrity. The resulting phenotype is progressive, combining both developmental shortening and degenerative arthropathy.

The mutations associated with FDAB exhibit a distinct site-specific pattern: all known pathogenic variants are located in the third finger loop and consistently display a loss-of-function effect. In contrast, mutations linked to other skeletal dysplasias and peripheral neuropathies lack clear positional specificity, with most reported variants in these disorders being predominantly gain-of-function. This case provides further evidence to support the association between *TRPV4* loss-of-function mutations and the FDAB phenotype, and also underscores the importance of precise functional characterization for genetic counseling and future therapeutic strategies, such as deciding whether to use agonists or antagonists.

## CRediT authorship contribution statement

**Xingzhu Geng:** Writing – review & editing, Writing – original draft, Funding acquisition. **Yunyun Zhang:** Writing – original draft, Data curation. **Chunhai Gao:** Writing – review & editing, Formal analysis, Conceptualization. **Kai Liu:** Writing – review & editing, Methodology. **Yuda Wei:** Writing – review & editing, Formal analysis. **Xiaxia Liu:** Writing – review & editing, Project administration. **Hongyan Xu:** Writing – review & editing, Methodology, Formal analysis. **Xiangyu Zhao:** Writing – review & editing, Supervision, Funding acquisition, Conceptualization.

## Ethics declaration

This project has been approved by the Ethics Committee of the Linyi People’s Hospital of Shandong Second Medical University (Ethics No. 202402-H-034). The patient provided informed consent for publication of the case.

## Funding

This work was supported by the 10.13039/501100001809National Natural Science Foundation of China (No. 82402155) and the 10.13039/501100007129Natural Science Foundation of Shandong Province, China (No. ZR2025QC207).

## Conflict of interests

The authors declared no conflict of interests.
