# Supplementary material for: Expanding the phenotypic spectrum of familial digital arthropathy-brachydactyly due to a novel monoallelic variant in TRPV4: A report of the fourth family
Source: Genes Dis. 2026 Mar 21;14(3):102158. doi: 10.1016/j.gendis.2026.102158 (PMC13734876; doi:10.1016/j.gendis.2026.102158)
Supplement: Supplementary file 1 [file mmc1.docx]

**Supplementary methods**

**Sample Collection and Processing**

Peripheral blood samples (4 ml each) were collected from the proband and family members via the cubital vein using EDTA anticoagulation. Genomic DNA was extracted from the peripheral blood using the Blood Genomic DNA Midiprep Kit , following the manufacturer's instructions. The DNA samples were quality-controlled using a Qubit 2.0 Fluorometer and 0.8% agarose gel electrophoresis, and stored at -20°C for high-throughput sequencing and Sanger sequencing verification.

**Whole Exome Sequencing (WES)**

Library Construction: The IDT xGen® Exome Research Panel v1.0 capture probes were used for liquid-phase hybridization with the gDNA library sequences to enrich target region DNA fragments, constructing the whole exome library. This library covers the coding regions and parts of non-coding regions of approximately 20,000 human genes, with a capture size of 51 Mb.

Sequencing: High-throughput sequencing (PE150) was performed using the Illumina NovaSeq 6000 series sequencer, achieving a target sequence coverage of no less than 99%. The sequencing process was completed by the Chigene Oriental Translational Medicine Research Center.

Bioinformatics Analysis: Fastp software was used for quality control of the raw data, removing adapters and low-quality reads. The Burrows-Wheeler Aligner (BWA) software was used to align the sequencing reads to the Ensembl reference genome GRCh37/hg19. GATK software was then used to identify SNPs and Indels. Finally, the detected SNPs and Indels were filtered and screened based on sequencing depth and mutation quality to obtain high-quality, reliable mutations. The detected variants were annotated by associating them with major databases (such as frequency databases like dbSNP, 1000 Genomes, ExAC, ESP, and databases like OMIM, HGMD, ClinVar). Their pathogenicity was analyzed using protein structure prediction software/tools such as Provean, SIFT, Polyphen2-HVAR, Polyphen2-HDIV, M-Cap, Revel, MutationTaster, and the MaxEntScan splice site prediction software, to screen for variants potentially having deleterious effects on protein structure.

**Sanger Sequencing**

Sanger sequencing was applied to validate the candidate pathogenic variant in the proband and family members. Primers were designed based on the sequence of the target site in the TRPV4 (NM_021625) gene. Amplification was performed using TAKARA's EX Taq enzyme. Primers are listed in Supplementary Table 1. The amplification products were sequenced using an ABI 3500 Genetic Analyzer, and sequence analysis was performed using Chromas software.

**Structure Modeling and Conservation Analysis**

The human TRPV4 structural model was generated based on the published cryo-EM structure of the human TRPV4 intracellular domain in complex with the GTPase RhoA (Protein Data Bank ID: 9IQY). All structural visualizations and analyses were performed using PyMOL (The PyMOL Molecular Graphics System, Version 2.6, Schrödinger, LLC). The missense mutation p.G270A was introduced in silico using the mutagenesis wizard in PyMOL. To evaluate potential structural alterations, residues within a 5 Å radius of the wild-type or mutant amino acid were identified, and local interactions (including hydrogen bonds) surrounding the substitution site were analyzed and visualized. Homologous protein sequences from multiple species were selected using NCBI protein database. Multiple sequence alignment was performed using the NCBI Multiple Sequence Alignment Viewer software (Version 1.25.3) to assess the conservation degree of specific amino acid sites during evolution.

**Supplementary Table 1** Primers for validation of candidate mutation sites

| Primers | Sequences | Annealing Temperature | Length of product |
| --- | --- | --- | --- |
| TRPV4-c.809G>C -F | CCCCAATTTCTACGTCCTGCACCCTA | 60 | 449bp |
| TRPV4-c.809G>C -R | GGTACTCCCCATGGCACTTACACC |  |  |
